# Splenic torsion with involvement of pancreas and descending colon in a 9-year-old boy

**DOI:** 10.1259/bjrcr.20180051

**Published:** 2018-06-27

**Authors:** Ali Seif Amir Hosseini, Ulrike Streit, Johannes Uhlig, Lorenz Biggemann, Fritz Kahl, Saheeb Ahmed, Duersch Markus

**Affiliations:** 1 Department of Diagnostic and Interventional Radiology, University Medical Center Göttingen, Göttingen, Germany; 2 Department of General-, Visceral-, and Pediatric Surgery, University Medical Center Göttingen, Göttingen, Germany

## Abstract

Splenic torsion is an uncommon condition becoming clinically apparent when the spleen twists or rotates around the organ’s vascular pedicle. In the case of a wandering spleen the organ is only attached to an elongated vascular pedicle while the peritoneal attachments are absent. However, splenic torsion could also occur in patients with abnormal laxity of the splenic peritoneal attachments. We report a case of a splenic torsion due to absence of splenic ligaments with pancreatic volvulus and partial involvement of descending colon in a 9-year-old boy.

## Clinical presentation and differential diagnosis

A 9-year-old boy presented with acute upper abdominal pain and loss of appetite. Symptoms persisted for over 2 days. Physical examination revealed a mobile abdominal mass. No prior history of chronic constipation and no history of abdominal trauma or prior surgery was reported by the parents. An initial abdominal ultra sound (US) revealed a well defined homogenous and echogenic mass of 21 cm average diameter, which was interpreted as splenomegaly in expected position with poorly, defined fluid in the surrounding tissues. US examination was limited by heavy meteorism, therefore, further imaging was indicated. Laboratory results showed normal level of platelet count of 156.0 × 10^3^ µl^−1^ (150.0–300.0), increased white blood cell count of 28.6 × 10^3^ µl^−1^ (4.5–13.5), increased LDH of 546.0 U l^−1^ (145.0–300.0) as well as increased CK of 3404.0 U l^−1^ (30.0–200.0) were pathologically elevated, though, liver function and renal function were normal.

## Imaging findings

**Figure 1.**
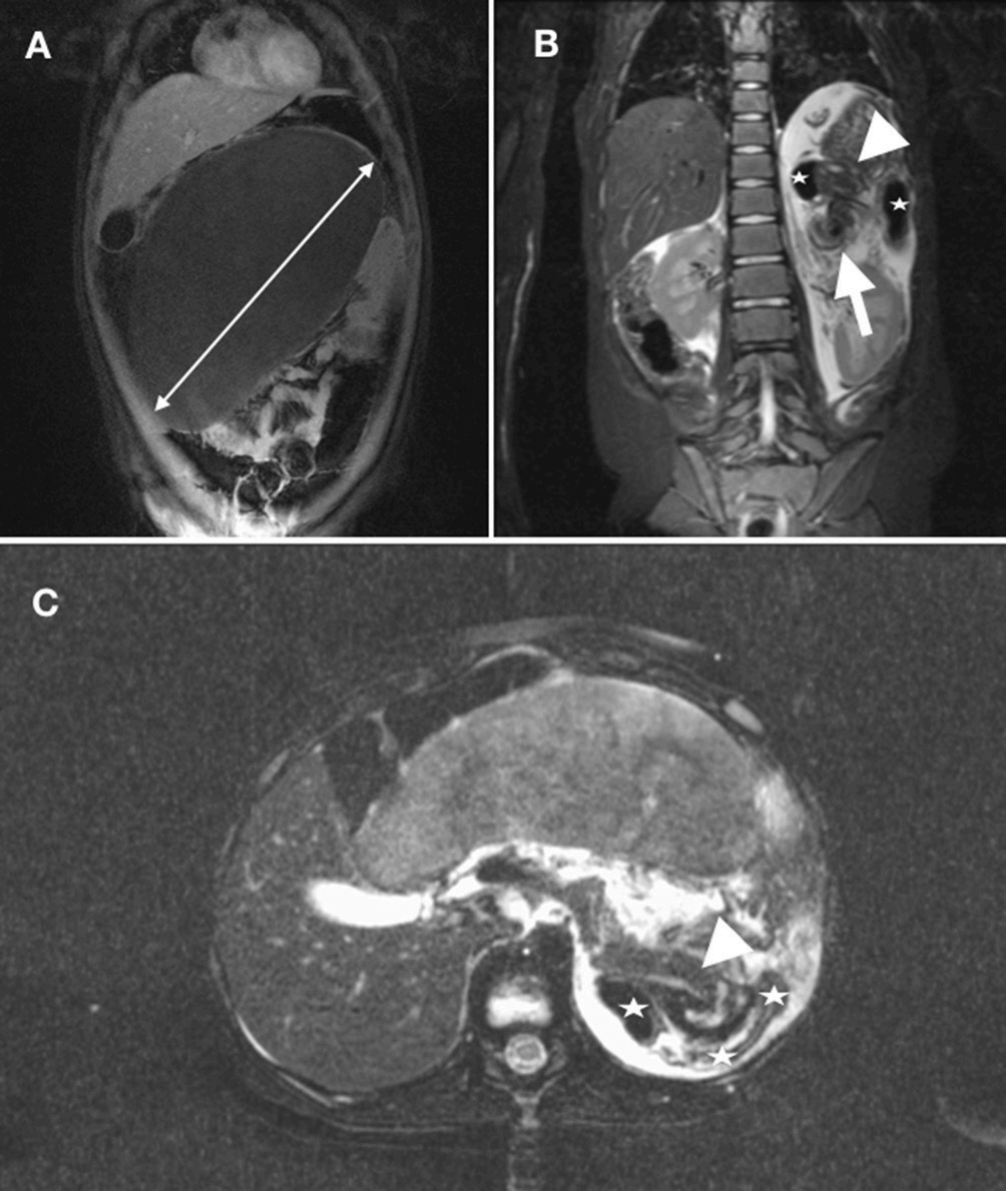
Contrast-enhanced *T*_1_ weighted MR-imaging (coronary view) reveals an enlarged, ischemic and translocated spleen towards the midline of the abdomen (A). *T*_2_ weighted and fat-saturated imaging (coronary view) shows the so-called “*whirl-sign*” (arrow) representing the torsion of the splenic pedicle (B). Involvement of neighboring anatomical structures such as pancreas (arrow head) and descending colon (star) is shown in (B) and (C). In the axial orientation (C) the arrow head points to a dilated main pancreatic duct.

Further diagnostics were conducted by magnetic resonance imaging (MRI) to assess the enlarged organ and other intraabdominal organs and to rule out malignancies. MRI revealed an enlarged and wedge-shaped spleen (Figure 1A and C). No enhancement could be detected after i.v. contrast administration. The vascular structures could be best depicted in the coronary orientation of the MRI with signs of anti clockwise twisting of the vascular pedicle (Figure 1B). The lack of enhancement in the *T*_1_ weighted study was interpreted as global splenic infarction. The splenic enlargement and a rather hyperintense *T*_2_ weighted signal was considered as a sign of edema and venous congestion due to the twisted vascular pedicle preventing blood drainage and subsequently leading to an enlargement of the spleen. There were also signs of poorly defined fluid in the surrounding tissues in the former splenic lodge as well as in the lower abdomen and pelvis.

**Figure 2.**
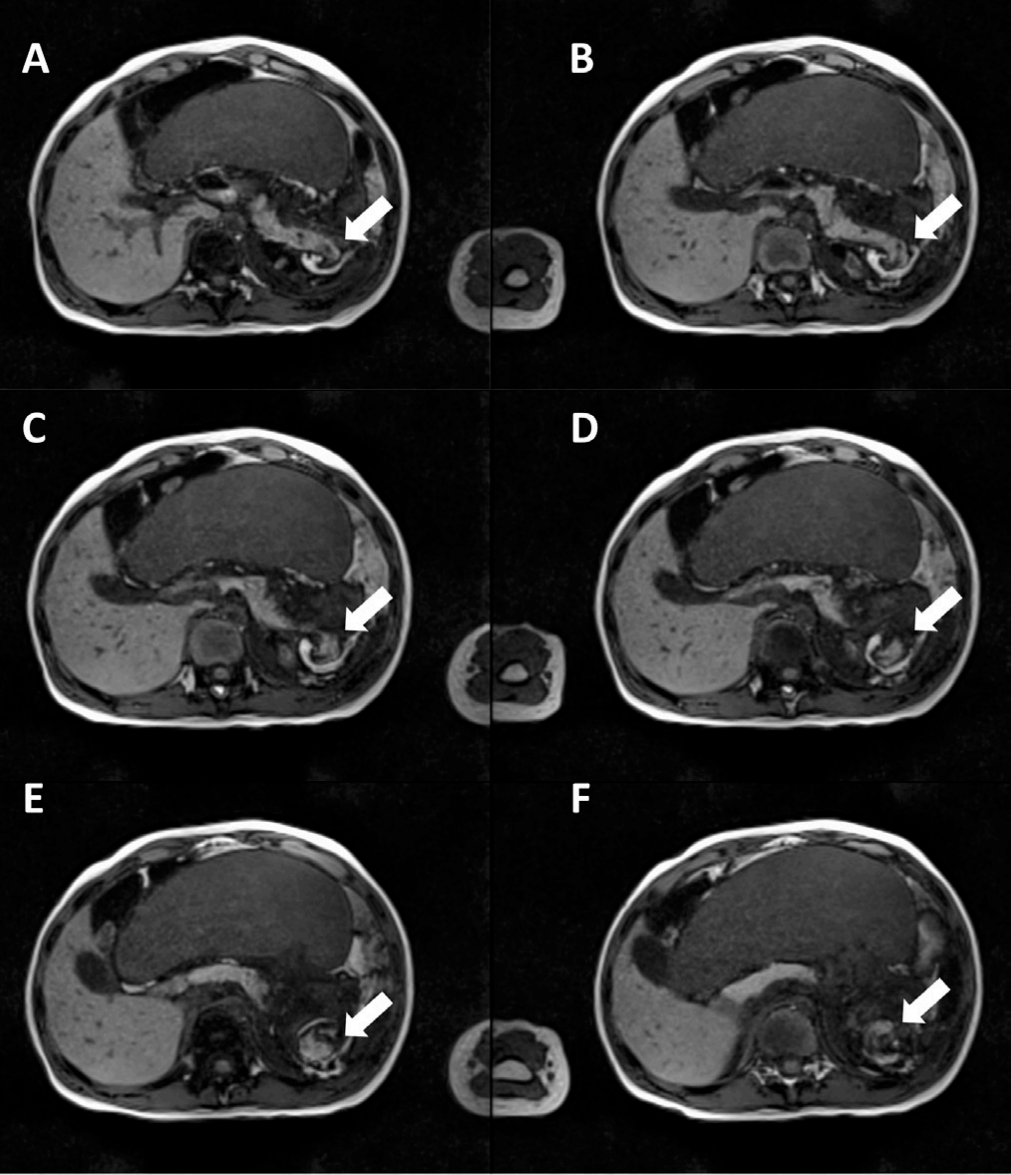
*T*_1_ weighted MR-imaging (transversal view) reveals the extent of the pancreatic involvement. (A-F) show the entrapment of the pancreatic tail (*“white arrow”*) in the twisted vascular pedicle during torsion of the spleen.

Another relevant finding revealed by the MRI was an involvement of the pancreas. MRI showed an involvement of the pancreatic tail with a focal dilatation of the pancreatic duct, suggesting an obstruction of the main pancreatic duct (Figure 1C). Further, MRI revealed kinking of the pancreatic tail (Figure 2A–F). No further pathological signal alterations of the pancreas were detectable. Also, MRI indicated an involvement of the descending colon, though, no signs of bowel obstruction were detectable.

## Treatment

**Figure 3.**
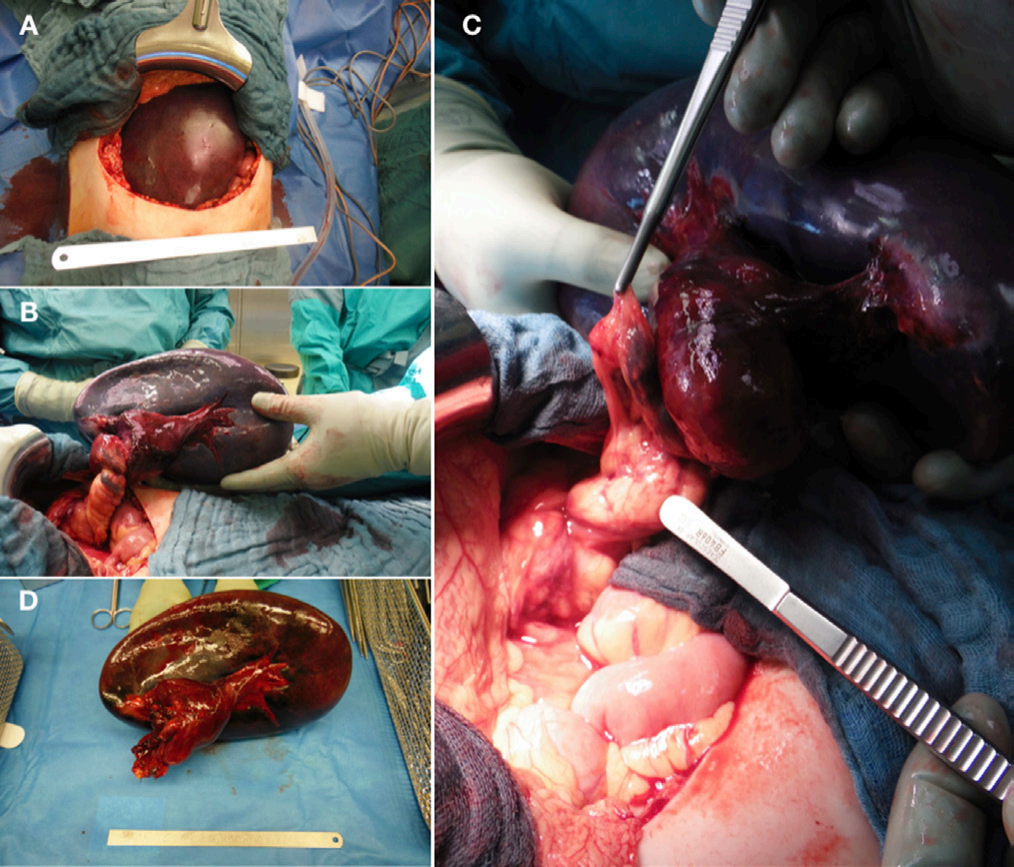
Upper transverse laparotomy showed a translocated spleen from the original location towards the midline of the abdomen directly underneath the abdominal wall (A). The splenic vascular pedicle was twisted several times with thrombosis and dilatation of the splenic artery and vein with a diameter of at least 1 cm. The twisted pedicle and the pancreatic tail are also involved (B, C). The spleen presented a diameter of 25 cm (D).

Emergent laparotomy was performed immediately after MRI examination. Access to the spleen was gained by an upper transverse abdominal laparotomy. The spleen was 25 cm in length and was translocated from the original location towards the midline of the abdomen directly underneath the abdominal wall (Figure 3A). All suspensor ligaments that usually hold the spleen in place in its compartment were absent.

The splenic vascular pedicle was twisted several times with thrombosis as well as dilatation of the splenic artery and vein with a diameter of at least 1 cm (Figure 3B and C). The organ showed substantial hemorrhagic infarction. Due to the translocation of the spleen, the pancreatic tail was kinked and twisted several times, as suggested by the MRI, though, no signs of pancreatic organ damage were detectable. Also, the suggested entrapment of the descending colon could be confirmed, however, there were no signs of damage of the descending colon. There was no acute abdominal bleeding with regular perfusion of the abdominal organs except for the spleen. The splenic torsion was resolved, however, no reperfusion was detectable. Due to the extensive infarction and congestion of the organ, the spleen could not have been preserved and splenectomy was performed.

The patient recovered fast after surgery with regular bowel movements from the second day after surgery and without any digestion problems. The boy was discharged on day 9 after surgery. He received a postoperative vaccination against Streptococcus pneumoniae, Haemophilus influenzae Type B and Neisseria meningitides on day 14 after splenectomy. The recovery period was uneventful and the follow-up examination after 2 months was inconspicuous.

## Discussion

Splenic torsion is a rather uncommon condition associated with the absence, underdevelopment or hyperlaxity of splenic suspensory ligaments, which causes an increased mobility of the spleen.^1–3^ In addition to that, a long vascular pedicle predisposes to acute or chronic intermittent torsion with or without infarction depending on whether congestion of the organ occurs or not. This condition is also referred to as “wandering spleen” with two peaks of incidence in children aged less than 10 years, as in this reported case, and in females of childbearing age.^1, 4,5^ This condition also accounts for 0.2–0.3% of all splenectomies.^3, 6,7^ The involvement of other organs are rather rare. Only a few cases reported a pancreatic involvement.^1, 2,8,9^ To the best of our knowledge there has been only one report about a splenic torsion with involvement of pancreas and descending colon.^1^

Patients with a wandering spleen can highly vary in their clinical presentation, ranging from being asymptomatic with an incidental palpable mass or present with an acute abdomen due to splenic infarction.^1, 3,6^ A splenic torsion can be a reason for recurrent abdominal pain, mostly localized in the left upper abdomen. The patients describe a colic pain lasting for a few moments to several hours with complete remission. Recurrent torsions with spontaneously detorsions of the spleen are in discussion for this rare clinical finding.^10, 11^

Imaging plays a major role in establishing the diagnosis. US with color-Doppler control may reveal an abnormal location of the spleen and detect a mass, usually located in the midline of the abdomen. US can deliver valuable information in the preoperative workup of splenic torsion, i.e. for the assessment of viability of the spleen, involvement of other organs and the “whirl sign”. Therefore, US should be considered as a primary diagnostic and preoperative imaging tool.^12^ However, the US examination depends highly on the examiner’s skills and it is often limited by meteorism.^9, 12^ In this presented case, US was inconclusive mainly due to heavy meteorism as described, and hence, further imaging with MRI was needed.

Tomographic examinations such as contrast-enhanced CT or MRI examinations may add valuable information in the course of diagnostic workup and can be included in case of an inconclusive or incomplete ultrasound examination. The most common findings include an empty splenic fossa and a translocated spleen. Additionally, in tomographic examinations the so-called “*whirl sign”* can be described, which is considered to be a sign of the torsion of the splenic pedicle.^12, 13^ Contrast-enhanced MRI is able to assess splenic viability. The above-mentioned pathology was found on MRI.

Surgical treatment options of wandering spleen depend on the organ’s viability. If the spleen shows proper reperfusion after resolving the splenic torsion, either open or laparoscopic splenopexy may be offered, due to the spleen’s physiologic importance, especially in children, and the risk of post-splenectomy sepsis.^14^ If, however, the spleen is substantially infracted, a partial subtotal resection or splenectomy should be considered.^1, 5^ After splenectomy it is recommended to perform vaccination against capsulated pathogens like pnemococcus, H influenzae and meningococcus.^15^

## Learning points

1. Splenic torsion with the involvement of neighboring anatomical structures is an uncommon condition.
2. However, it should be considered in differential diagnosis of an acute abdomen, especially in pediatric patients.
3. Therefore, accurate preoperative imaging is mandatory. Contrast-enhanced MRI is a suitable method to deliver valuable preoperative information regarding the spleen’s viability and the possible involvement of other neighboring organs. However, due to limited availability of MRI, US should be the first choice in the diagnostic path.

## 

## References

[1] Flores-Ríos E, Méndez-Díaz C, Rodríguez-García E, Pérez-Ramos T Wandering spleen, gastric and pancreatic volvulus and right-sided descending and sigmoid colon. J Radiol Case Rep 2015; 9: 18–25. doi: 10.3941/jrcr.v9i10.2475 PMC4638397 26629290

[2] Gorsi U, Bhatia A, Gupta R, Bharathi S, Khandelwal N Pancreatic volvulus with wandering spleen and gastric volvulus: an unusual triad for acute abdomen in a surgical emergency. Saudi J Gastroenterol 2014; 20: 195–8. doi: 10.4103/1319-3767.133026 24976284 PMC4067917

[3] Raissaki M, Prassopoulos P, Daskalogiannaki M, Magkanas E, Gourtsoyiannis N Acute abdomen due to torsion of wandering spleen: CT diagnosis. Eur Radiol 1998; 8: 1409–12. doi: 10.1007/s003300050562 9853224

[4] Liu HT, Lau KK Wandering spleen: an unusual association with gastric volvulus. AJR Am J Roentgenol 2007; 188: W328–W330. doi: 10.2214/AJR.05.0672 17376999

[5] Priyadarshi RN, Anand U, Kumar B, Prakash V Torsion in wandering spleen: CT demonstration of whirl sign. Abdom Imaging 2013; 38: 835–8. doi: 10.1007/s00261-012-9944-9 22829098

[6] Ben Ely A, Zissin R, Copel L, Vasserman M, Hertz M, Gottlieb P, et al The wandering spleen: CT findings and possible pitfalls in diagnosis. Clin Radiol 2006; 61: 954–8. doi: 10.1016/j.crad.2006.06.007 17018308

[7] Eraklis AJ, Filler RM Splenectomy in childhood: a review of 1413 cases. J Pediatr Surg 1972; 7: 382–8. doi: 10.1016/0022-3468(72)90006-1 5049847

[8] Aswani Y, Anandpara KM, Hira P Wandering spleen with torsion causing pancreatic volvulus and associated intrathoracic gastric volvulus. An unusual triad and cause of acute abdominal pain. JOP 2015; 16: 78–80. 25640790 10.6092/1590-8577/2905

[9] Sheflin JR, Lee CM, Kretchmar KA Torsion of wandering spleen and distal pancreas. AJR Am J Roentgenol 1984; 142: 100–1. doi: 10.2214/ajr.142.1.100 6606938

[10] Jha AK, Ranjan R, Priyadarshi RN, Spleen W Wandering spleen and portal h\Hypertension: a vicious interplay. ACG Case Rep J 2017; 4: e54. doi: 10.14309/crj.2017.54 28459078 PMC5404629

[11] Tan HH, Ooi LL, Tan D, Tan CK Recurrent abdominal pain in a woman with a wandering spleen. Singapore Med J 2007; 48: e122–4. 17384868

[12] Peretti M, Mariotto A, Scirè G, Pani E, Zambaldo S, Bianchi S, et al Wandering spleen with a ten-time twisted vascular pedicle. Pediatr Med Chir 2016; 38: 119. doi: 10.4081/pmc.2016.119 28009140

[13] Fonseca AZ, Ribeiro M, Contrucci O Torsion of a wandering spleen treated with partial splenectomy and splenopexy. J Emerg Med 2013; 44: e33–e36. doi: 10.1016/j.jemermed.2011.06.146 22381612

[14] Cohen MS, Soper NJ, Underwood RA, Quasebarth M, Brunt LM Laparoscopic splenopexy for wandering (pelvic) spleen. Surg Laparosc Endosc 1998; 8: 286–90. doi: 10.1097/00019509-199808000-00010 9703603

[15] Buzelé R, Barbier L, Sauvanet A, Fantin B Medical complications following splenectomy. J Visc Surg 2016; 153: 277–86. doi: 10.1016/j.jviscsurg.2016.04.013 27289254

